# Assessment of Salinomycin’s Potential to Treat *Microcotyle sebastis* in Korean Rockfish (*Sebastes schlegelii*)

**DOI:** 10.3390/ani13203233

**Published:** 2023-10-17

**Authors:** Won-Sik Woo, Sang Hee Shim, Gyoungsik Kang, Kyung-Ho Kim, Ha-Jeong Son, Min-Young Sohn, Seungjin Lee, Jaekyeong Kim, Jung-Soo Seo, Mun-Gyeong Kwon, Do-Hyung Kim, Chan-Il Park

**Affiliations:** 1 Department of Marine Biology & Aquaculture, Institute of Marine Industry, College of Marine Science, Gyeongsang National University, 2 Tongyeonghaean-ro, Tongyeong 53064, Republic of Korea; 2 Natural Products Research Institute, College of Pharmacy, Seoul National University, Seoul 08826, Republic of Korea; 3 Aquatic Disease Control Division, National Fishery Products Quality Management Service, 216 Gijanghaean-ro, Busan 46083, Republic of Korea; 4 Department of Aquatic Life Medicine, College of Fisheries Sciences, Pukyong National University, Busan 48513, Republic of Korea

**Keywords:** salinomycin, *Sebastes schlegelii*, *Microcotyle sebastis*, anthelmintic, parasites, monogenean

## Abstract

**Simple Summary:**

Aquaculture is vital for global food production, but parasites like *Microcotyle sebastis* pose challenges to Korean rockfish, leading to economic concerns. As there is a growing worry about drug resistance, we explored salinomycin, previously recognized for treating other parasites, to combat this issue. Our experiments revealed that salinomycin effectively lessens these parasites in Korean rockfish without significant side effects. Importantly, the treatment appeared potentially more stable when administered at a water temperature of 13 °C. This study suggests that salinomycin could be a promising alternative, especially if resistance against current treatments like praziquantel emerges.

**Abstract:**

Aquaculture, a crucial sector of the global food industry, faces a myriad of issues due to parasitic invasions. One such parasite, *Microcotyle sebastis*, which afflicts Korean rockfish in South Korea, has a significant economic impact. The impending danger of resistance to traditional anthelmintics necessitates the exploration of new antiparasitic candidates. Although the efficacy of salinomycin against aquatic parasites such as ciliates and sporozoans is known, its influence on monogeneans has yet to be studied. Therefore, this study investigated the efficacy and safety of salinomycin for the treatment of *M. sebastis* infections, presenting the first exploration of salinomycin’s therapeutic potential against monogeneans. In vitro examinations revealed a minimum effective concentration of salinomycin of 5 mg/kg, which led to necrosis of the haptor upon dislodging from the gill filaments. The one-time oral administration of the drug at concentrations of 5 mg/kg and 10 mg/kg showed a significant dose-dependent reduction in parasite counts, with no apparent behavioral side effects in Korean rockfish. Biochemical analyses monitored the liver, heart, and kidney enzymes, specifically aspartate transaminase (AST), alanine transaminase (ALT), blood urea nitrogen (BUN), and creatine kinase–myocardial band (CK-MB). At both 20 °C and 13 °C, no significant differences were observed in the levels of AST and ALT. However, at 20 °C, alterations in BUN levels were evident on Day 14, a deviation not observed at 13 °C. The CK-MB analysis revealed elevated enzyme levels at both temperatures when compared to the control group, reflecting the similar changes observed in terrestrial animals administered salinomycin. The biochemical data suggest that the oral administration of salinomycin is potentially more favorable at 13 °C than at 20 °C. Although our findings warrant further comprehensive studies, including on the long-term and potential effects on nontarget species and water quality, they also suggest that salinomycin could be considered as an alternative or adjunctive treatment if resistance to the currently used praziquantel against *M. sebastis* is confirmed.

## 1. Introduction

Aquaculture is an increasingly important sector of the global food industry, providing a significant source of protein for millions of people worldwide [1]. However, it faces numerous challenges, including the detrimental impact of parasites on fish health and production [2]. These parasites inflict various forms of damage, leading to stunted growth, elevated mortality rates, compromised immunity, behavioral alterations, and considerable economic losses [3]. In particular, infections of Korean rockfish (*Sebastes schlegelii*) by the gill parasite *Microcotyle sebastis* are observed throughout the year [4]. However, these infections predominantly occur en masse around the summer, when water temperatures approach 20 °C [5,6]. Infection rates ranging from a minimum of 46.7% to a maximum of 96.7% have been reported [7]. This proliferation can cause respiratory distress leading to mortality, or excessive inflammation and mucus secretion, which in turn can result in secondary infections or stunted growth, posing challenges in aquaculture settings [8].

The infection process of *M. sebastis* in Korean rockfish involves several stages. Following the hatching of eggs, the adult parasites give rise to free-swimming oncomiracidium [9]. Utilizing both the flow of seawater and the respiration of the host, these organisms attach themselves to the gill filaments of the fish [10]. Praziquantel has served as the principal therapeutic option for treating *M. sebastis* infections in Korean rockfish for approximately 20 years [11]. However, emerging reports have suggested a potential resistance to praziquantel in several monogenean species [12,13]. This necessitates the exploration and development of alternative anthelmintics, given the documented decline in susceptibility across various parasite species [14,15,16,17].

Salinomycin, a polyether antibiotic, has been extensively utilized in the poultry industry as an anticoccidial agent and a growth promoter for ruminant animals [18,19]. The diverse pharmacological properties of salinomycin, such as its capacity to disrupt cellular ion balance and its selective toxicity against cancer stem cells, position salinomycin as a promising candidate for research into applications against various pathogens [20,21]. Moreover, documented antiparasitic effects span a range of parasites, including protozoa, cestodes, and flagellates [22,23,24,25,26]. The primary antiparasitic action of salinomycin is attributed to its ability to form monovalent cation (especially K+) and lipophilic complexes, which subsequently promote transmembrane cation transport and increase osmotic pressure in coccidia [27]. Additionally, salinomycin’s interactions with multiple biological membranes, especially cytoplasmic and mitochondrial ones, can lead to mitochondrial damage and the suppression of mitochondrial oxidative phosphorylation, ultimately resulting in cellular death [21]. However, in the absence of research concerning the efficacy of salinomycin against monogeneans such as *M. sebastis*, it is imperative to conduct research to validate the potential applicability of salinomycin’s anti-parasitic action for these parasite species.

In aquaculture, common drug administration methods include injection, immersion, and oral administration [28,29]. Determining the efficacy and safety of these methods is essential [30,31,32]. For fish species farmed in sea cages, injection is often impractical due to space constraints [33]. Currently, immersion is the primary method used [34]. However, this technique necessitates a large quantity of medication relative to its therapeutic effect and demands significant labor for drug preparation [35]. In contrast, oral administration emerges as a promising alternative [36]. By simply incorporating the drug into feed, this method offers a way to achieve therapeutic effects with batch feeding. Not only does oral administration reduce stress on the fish, but it also requires less labor [36]. However, its efficacy might be affected by digestive enzymes in the gastrointestinal tract. The actual therapeutic outcome can also vary depending on the feed intake [37]. Notably, the majority of research on salinomycin application in aquaculture has centered on its efficacy when administered orally [23,25,38].

In vitro susceptibility testing is foundational in determining the potential of therapeutic pharmaceuticals. Determining the appropriate drug concentration and its anticipated effects is crucial before progressing to more intricate in vivo studies [39]. These in vivo tests, when supplemented with in vitro results, shed light on a drug’s efficacy, metabolism, and safety in a living organism. Blood biochemical analyses are equally critical, providing insights into the physiological and metabolic reactions of the organism to the therapeutic, thereby ensuring its safety and efficacy [40,41,42]. An integrated approach, amalgamating in vitro, in vivo, and biochemical studies, is pivotal to ascertaining whether potential therapeutics are both efficacious and safe for wider applications, such as in aquaculture. This comprehensive testing approach not only protects the target species but also safeguards the broader ecosystem, reducing the risk of unintended ecological impacts or the emergence of drug-resistant parasites [43].

This study aims to ascertain whether salinomycin exhibits effective anthelmintic activity against *M. sebastis* in vitro and to assess the safety and efficacy of orally administered salinomycin in the treatment of *M. sebastis* infections in Korean rockfish. Our investigation encompasses the validation of *M. sebastis*’s susceptibility to salinomycin in an in vitro environment, an evaluation of the efficacy of oral administration in vivo, and an assessment of treatment safety via post-administration biochemical analyses at both 20 °C and 13 °C.

Through this comprehensive approach, we seek to provide robust insights into the potential of salinomycin as an effective anthelmintic agent in the context of aquaculture, particularly focusing on its potential for mitigating the detrimental effects of *M. sebastis* infections in Korean rockfish populations. The findings from this study are expected to offer valuable contributions towards the development of improved parasitic control strategies in aquaculture settings, potentially enhancing productivity and economic viability within the industry.

## 2. Materials and Methods

### 2.1. Fish

For both in vitro and in vivo experiments, Korean rockfish were procured from a farm near Tongyeong City, Gyeongsangnam-do. The fish were then euthanized using a solution of MS222 powder (Sigma-Aldrich, St. Louis, MO, USA) dissolved in 100% ethanol to achieve a final concentration of 500 mg/L [44]. Subsequent to this, the presence of *M. sebastis* infection was confirmed in the gills. The fish used in the study had a mean length of 15.5 cm with a standard deviation (SD) of ±2 cm and a mean weight of 37.8 g with an SD of ±3.5 g. A total of 350 Korean rockfish were acclimated over a period of one week in a 2.5-ton tank maintained at a temperature of 20.3 ± 0.4 °C. During this period, the dissolved oxygen (DO) levels were maintained at 5.0 ± 0.3 mg/L, and salinity was held at 34.1 ± 0.22‰. A daily water exchange of 50% was conducted, encompassing both the main tank and the pre-entry tank.

### 2.2. In Vitro Assessment of M. sebastis Sensitivity to Salinomycin

*M. sebastis* was harvested from Korean rockfish euthanized using MS222 solution at a concentration of 500 mg/L. The gills of the Korean rockfish were carefully removed, and at room temperature, any mucus and contaminants were gently eliminated by briefly dipping them in sterile seawater for no more than 10 s per dip, repeating this three times. *M. sebastis*, delicately extracted while remaining attached to the gill filaments, were collected using forceps and dissecting scissors. Subsequently, to facilitate easier identification in subsequent in vitro experiments, the strands to which *M. sebastis* were attached were individually separated from the gill using fine forceps and dissecting scissors. These parasites, still attached to the gill filament strands, were transferred to a Petri dish filled with sterile seawater and allowed to acclimate for one hour before in vitro experimentation.

The sensitivity of *M. sebastis* to salinomycin (Sigma-Aldrich, USA) was assessed in a 12-well plate containing 2 mL of sterile seawater in each well. For the assessment, 20 individuals of *M. sebastis* were allocated to each of the five experimental wells, one of which served as a control group. Salinomycin was dissolved using dimethyl sulfoxide (Sigma-Aldrich, USA) and then added to each well plate at concentrations of 2.5, 5, 7.5, and 10 mg/L. Changes in the parasites, both before and after exposure to salinomycin, were documented using an inverted microscope ECLIPSE Ts2 (Nikon, Tokyo, Japan). The behavior of the parasites, while adhered to the gill filaments, was observed at intervals of 1, 2, 3, 6, 9, and 12 h. Photographs captured using the inverted microscope were subsequently analyzed with the Image J 1.53t software to calculate and annotate with a scale bar [45]. The minimum effective concentration (MEC) was determined based on the exposure time and the point at which the parasites were considered dead. Mortality was defined as the absence of movement in *M. sebastis* and the lack of observable activity in the internal organs. The experiment was conducted in triplicate.

### 2.3. In Vivo Assessment of M. sebastis Efficacy to Salinomycin

The therapeutic potential of salinomycin was evaluated in Korean rockfish with *M. sebastis* infections. The adopted approach to parasite quantification was built upon established methods from prior monogenean studies [46]. To facilitate the examination, Korean rockfish with *M. sebastis* infections were anesthetized using MS222 solution at a dose of 100 mg/L [47]. Subsequently, individual gills were meticulously inspected with forceps and tweezers, and we conducted a precise enumeration of the adult *M. sebastis* parasites present in each culture tank. Enumeration was undertaken based on individuals of a size that could be visually confirmed. All Korean rockfish involved in the study had between 5 and 10 *M. sebastis*. Separately, the omega-3 fatty acid (Kirkland, QC, Canada) was sourced from commercially available gel capsules. The choice to use omega-3 fatty acids in this study was underpinned by their dual benefits. Firstly, their lipid properties facilitate the uniform distribution of the hydrophobic salinomycin, ensuring consistent dosing. Secondly, given that omega-3 is a standard component in fish feed, its incorporation can help assess the potential for the oral feeding of salinomycin. Using dissection scissors, a small incision was made in the capsules to extract the contained liquid, which was then transferred to a 50 mL corning tube. This omega-3 liquid was subsequently combined with the salinomycin solution, ensuring an appropriate mixture based on the intended experimental concentrations of 5 mg/kg and 10 mg/kg. The combined solution was thoroughly mixed to ensure homogeneous distribution. The final formulation was designed to achieve the intended dose for each experimental group with a 100 uL volume, which was administered as a single dose to the fish based on their body weight.

For the in vivo experiments, four tanks, each with a capacity of 120 L, were utilized. Specifically, two tanks were designated for the two salinomycin concentrations: one for the 5 mg/L dosage and the other for the 10 mg/L dosage. The remaining two tanks served as control groups. Each tank housed 20 Korean rockfish. The oral administration protocol was adopted from prior research designed to minimize reflux during the oral delivery of anthelmintics [48]. The prepared salinomycin solution (5 mg/L, 10 mg/L) was loaded into a syringe connected to an oral zonde (5 cm, Ø0.9 × 50 mm). Anesthetized fish were gently held in a vertical position with hands pre-wetted in seawater, and the solution was administered according to each experimental concentration. The control group was administered both the PBS solution and omega-3 using the same method to examine any potential effects of oral administration on the fish. All test groups were consistently monitored for behavioral changes and mortality.

Since *M. sebastis* eggs begin to hatch 8 days after initial spawning at a temperature of 20 °C, a cautious approach was adopted for efficacy evaluation [49]. More specifically, taking into account the extended time required for the drug to reach *M. sebastis* through oral administration, anthelmintic efficacy was assessed on the seventh day post-administration. During these 7 days, the fish in the oral administration group were maintained in a seawater recirculation filtration system with a salinity of 35.2 ± 0.5‰ at 20 °C, receiving daily rations equivalent to 0.2% of their body weight and undergoing 50% water replacement every day.

Then, 7 days after oral administration, the Korean rockfish were euthanized with an excess of MS222 solution at a concentration of 500 mg/L. The gills were extracted and placed in separate Petri dishes filled with sterilized seawater. The numbers of *M. sebastis* attached to the gill filaments were counted visually, and a stereomicroscope was utilized to further confirm the presence of smaller *M. sebastis*.

### 2.4. Biochemical Analysis

Based on the evaluation of in vivo efficacy, a blood biochemistry analysis for safety assessment was conducted using Korean rockfish under conditions similar to those described in our previous in vivo experiments [46]. All Korean rockfish were infected with *M. sebastis*, and six tanks were installed for both experimental and control groups, each with a capacity of 120 L and able to accommodate 35 Korean rockfish. The water temperature was initially set and maintained at 13 °C, reflecting the average temperature during the initial confinement culture of the Korean rockfish, and at 20 °C, where damage occurs due to *M. sebastis* infection, to confirm stability under these conditions. Two experimental groups and one control group were assigned to each temperature. The conditions in all tanks were consistently maintained at a salinity of 34.2 ± 0.5‰ and DO levels of 5.0 ± 0.3 mg/L. The experimental groups received the oral administration of salinomycin solution at concentrations of 5 mg/kg and 10 mg/kg, prepared in the same manner as in previous in vivo studies, while the control groups received a corresponding volume of omega-3.

On days 1, 3, 7, 14, and 28 after administration, six Korean rockfish from each group were chosen for sample collection. Blood samples were extracted from the caudal vein using a 1 mL syringe, then placed into Eppendorf tubes and preserved at 4 °C until the following day. The serum was extracted via centrifugation at 3000 rpm for 20 min and stored at −80 °C until analysis. Biochemical analyses were carried out on the obtained serum to assess the potential hematological effects of the anthelmintics on the Korean rockfish. The analysis included measurements of aspartate transaminase (AST), alanine transaminase (ALT), blood urea nitrogen (BUN) and creatine kinase–myocardial band (CK-MB) using a FUJI DRI-CHEM 4000I device, as per the manufacturer’s instructions.

### 2.5. Statistical Analysis

Statistical analysis was performed using GraphPad Prism software, version 9.5.1. Sensitivity to salinomycin in vitro was assessed for significance using the Friedman test. The anthelmintic efficacy of salinomycin was evaluated in vivo by comparing parasite counts before and after treatment in the experimental groups. The efficacy evaluation and biochemical analysis were both expressed as mean ± SD, assuming a normal distribution as determined by the Shapiro–Wilk test for normality. Statistical significance for the efficacy evaluation and biochemical analysis was determined using two-way analysis of variance (ANOVA), followed by post hoc testing with the Tukey test. Statistical significance levels were indicated as follows: * *p* < 0.05; ** *p* < 0.01; *** *p* < 0.001; **** *p* < 0.0001.

## 3. Results

### 3.1. In Vitro Assessment of M. sebastis’ Sensitivity to Salinomycin

**Figure 1:**
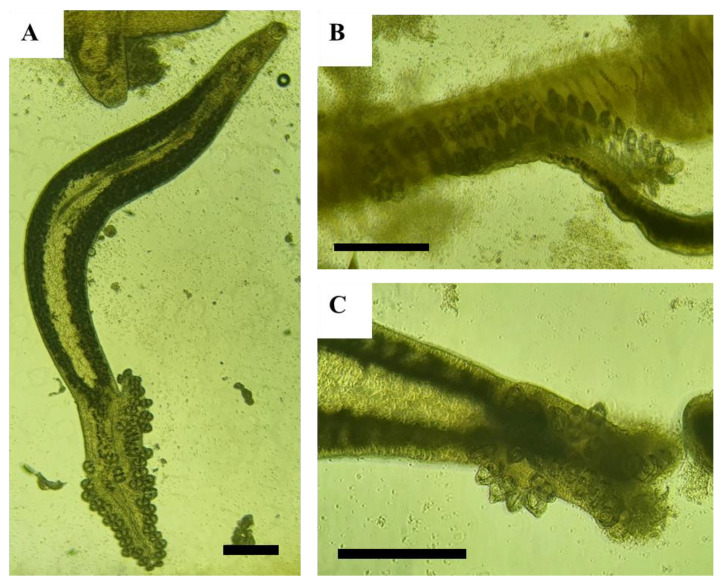
Microscopic images of *Microcotyle sebastis* from an in vitro assessment. (**A**) shows the overall appearance of *M. sebastis* without exposure to the drug, (**B**) depicts a control specimen of *M. sebastis* attached to gill filaments, and (**C**) presents the haptor of a *M. sebastis* individual that died as a result of exposure to salinomycin. The scale bar, represented by the black bar, corresponds to a length of 500 µm.

Exposure to salinomycin resulted in the detachment and necrosis of the posterior attachment organs of *M. sebastis* in gill filaments (Figure 1).

**Figure 2:**
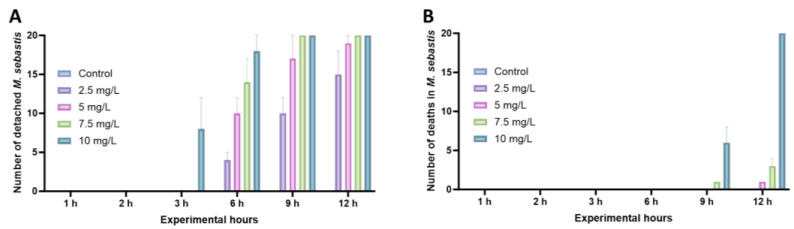
Detachment and deaths of *Microcotyle sebastis* exposed to salinomycin in vitro. (**A**) represents the number of *M. sebastis* that detached from the gill filaments following exposure to salinomycin, while (**B**) indicates the number of *M. sebastis* that died post-exposure to salinomycin.

In contrast to the control group, where no detachment or death was observed in the gill filaments, 8 out of 20 parasites (40%) were dislodged from the gill filaments 3 h after exposure to a salinomycin concentration of 10 mg/L. By the 9 h mark post-exposure, all *M. sebastis* were detached from the gill filaments (Figure 2A). Detachment from the gill filaments was observed from the 6th hour onwards at concentrations of 7.5 mg/L, 5 mg/L, and 2.5 mg/L, with an average of 14, 10, and 4 individuals, respectively. The lowest concentration at which mortality was observed was 5 mg/L, with an average of one death observed 12 h after exposure (Figure 2B). A gradual increase in death manifested in a concentration-dependent manner, with one and six deaths observed at 9 h post-exposure at concentrations of 7.5 mg/L and 10 mg/L, respectively, and total death occurring at 12 h at 10 mg/L, in contrast to three deaths at 7.5 mg/L. Based on these in vitro results, the MEC exhibiting death at the lowest concentration, 5 mg/L, was determined for subsequent in vivo experiments.

### 3.2. In Vivo Assessment of M. sebastis Efficacy to Salinomycin

**Figure 3:**
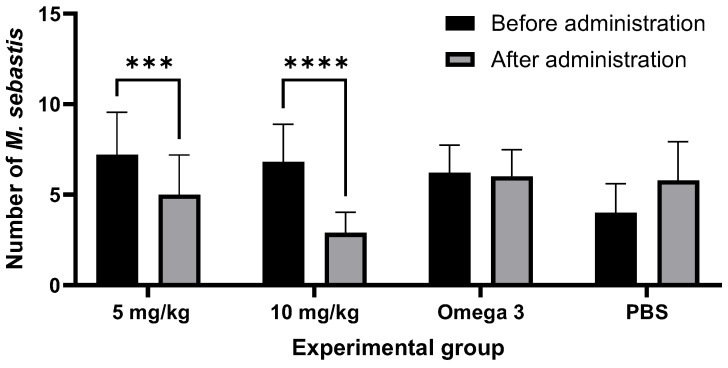
The anthelmintic efficacy of salinomycin against *Microcotyle sebastis* following oral administration. Significant differences are denoted by the respective superscripts (*** *p* < 0.001; **** *p* < 0.0001).

The in vivo study was conducted to evaluate the efficacy of salinomycin after a single oral administration, based on the MEC determined in the in vitro experiments (Figure 3).

Korean rockfish administered with a salinomycin solution did not exhibit any behavioral signs of stress or mortality throughout the experimental period. Efficacy was demonstrated by a reduced parasite count on day 7 post-administration in groups treated with doses of 5 mg/kg or 10 mg/kg, compared to the control group (*p* = 0.9946, 0.5589). No significant changes in the count of *M. sebastis* were observed in the omega-3 and PBS experimental control groups. A 31% reduction in the parasite count from an initial 7.2 ± 2.3 to 5 ± 2.1 was observed in the 5 mg/kg group after 7 days (*p* = 0.0008), while in the 10 mg/kg group, there was a 57% reduction from an initial 6.8 ± 2 to 2.9 ± 1.1 (*p* < 0.0001). The difference in the anthelmintic-treated groups was statistically significant, and the anthelmintic efficacy was confirmed to be dose-dependent. No mortalities were observed, and no abnormal symptoms were evident immediately after administration or during the 7-day observation period. The safety of salinomycin was subsequently confirmed through the biochemical analysis of blood.

### 3.3. Biochemical Assessment of Salinomycin’s Safety

**Figure 4:**
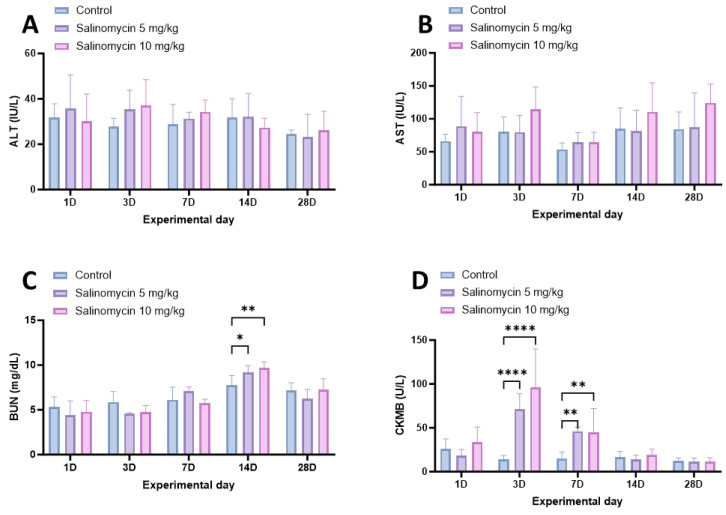
Biochemical analysis of Korean rockfish post-oral administration of varying concentrations of salinomycin at 20 °C. (**A**) Levels of alanine transaminase (ALT), (**B**) levels of aspartate transaminase (AST), (**C**) levels of blood urea nitrogen (BUN), and (**D**) levels of creatine kinase–myocardial band (CK-MB). Significant differences are denoted by respective superscripts (* *p* < 0.05; ** *p* < 0.01; **** *p* < 0.0001).

**Figure 5:**
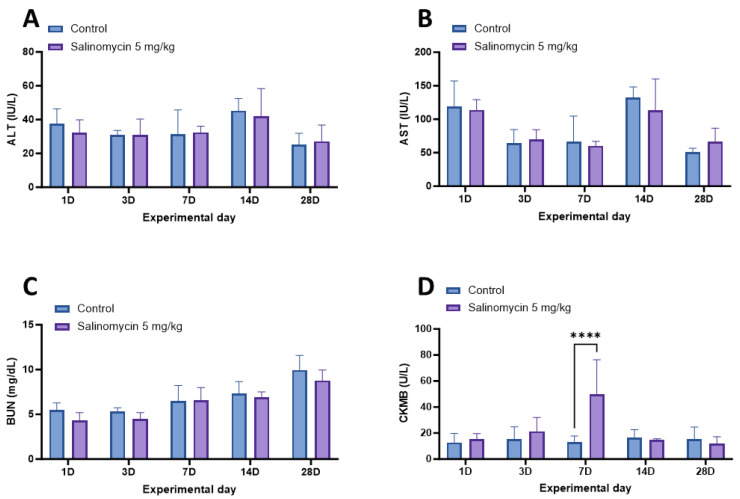
Biochemical analysis of Korean rockfish post-oral administration of salinomycin at 13 °C. (**A**) Levels of alanine transaminase (ALT), (**B**) levels of aspartate transaminase (AST), (**C**) levels of blood urea nitrogen (BUN), and (**D**) levels of creatine kinase–myocardial band (CK-MB). Significant differences are denoted by respective superscripts (**** *p* < 0.0001).

To evaluate the safety of salinomycin in Korean rockfish, serum samples were collected from six fish at 1, 3, 7, 14, and 28 days after the oral administration of salinomycin at various concentrations and temperatures. Subsequently, the levels of AST, ALT, BUN, and CK-MB were analyzed (Figure 4 and Figure 5). The Korean rockfish administered with a salinomycin solution did not exhibit any behavioral signs of stress or mortality throughout the experimental period. In the analysis of AST and ALT carried out after oral administration at 20 °C, both the 5 mg/kg and 10 mg/kg treatment groups showed no significant difference (Figure 4A,B). For the BUN measurements, distinct variations were evident on day 14 for both the 5 mg/kg (*p* = 0.442) and 10 mg/kg (*p* = 0.0036) dose groups (Figure 4C). Regarding the CK-MB evaluations, the 5 mg/kg and 10 mg/kg dose groups both showed marked deviations when juxtaposed with the control group on days 3 (*p* < 0.0001) and 7 (*p* = 0.0019, *p* = 0.0027). However, no significant differences were observed between the treatment and control groups after day 14 (Figure 4D).

In the AST, ALT, and BUN analyses conducted after oral administration at 13 °C, no significant difference was detected between the control group and the treatment groups (Figure 5A–C). However, at 13 °C, the analysis of CK-MB concentration showed a statistically significant difference in the 5 mg/kg treatment group compared to the control group on day 7 (*p* < 0.0001; Figure 5D).

## 4. Discussion

Research on using salinomycin, a treatment for coccidiosis in chickens, in aquaculture to treat parasites has predominantly focused on ciliates and myxozoans [22,23,25]. To the best of our knowledge, this study is the first to apply salinomycin to monogeneans. *M. sebastis*, a gill parasite of the Korean rockfish, starts its parasitic life cycle when a free-swimming larva, known as an oncomiracidium, attaches to the gill filaments using its haptor [9]. These haptors are vital for firmly gripping to the host’s gill filaments, preventing detachment, facilitating growth, and increasing in number, playing a pivotal role throughout *M. sebastis*’s life stages [9]. Our in vitro observations revealed that the haptors of *M. sebastis* were damaged upon exposure to salinomycin, leading to necrosis and detachment from the gill filaments. Once detached from the gills, these parasites face environmental threats and cannot feed, limiting their reproductive capabilities. A significant concern in aquaculture is complications arising from the unchecked proliferation of such parasites, which can cause gill anemia and respiratory challenges in host species [50]. Managing these parasite populations effectively is imperative. Our research suggests that salinomycin offers potential efficacy in addressing these challenges. However, since the anthelmintic action of salinomycin is primarily documented for species like coccidiosis, more research is needed to explore its activity against monogeneans.

In our in vivo studies, single doses of 5 mg/kg and 10 mg/kg demonstrated efficacies of 31% and 57% against *M. sebastis*, respectively. At first glance, these percentages might seem to indicate modest efficacy. However, the importance of these findings becomes evident when considering the limitations of single-dose administration and the lower bounds of effective concentrations. Our study aimed to eliminate parasites without causing significant harm to the host fish. High doses of anthelmintics can cause undue stress in aquaculture populations and disrupt innate immune responses, highlighting the im-portance of careful dosing [51]. Anthelmintic application in aquaculture usually involves bathing or oral administration [52]. Notably, oral administration often requires prolonged treatment durations. For instance, studies targeting the control of *Cryptocaryon irritans*, a parasitic threat to *Paralichthys olivaceus*, confirmed enhanced survival rates when salinomycin was orally given at a concentration of 200 ppm over two weeks [23]. Based on this evidence, our findings corroborate the hypothesis that the efficacy of salinomycin is dose-dependent, laying the groundwork for potential applications involving increased salinomycin concentrations in future investigations.

Enzymes are instrumental in assessing farmed fish health through biochemical analysis post drug treatment [53,54]. AST and ALT are primarily found in the blood and are associated with liver function, myocardial, and muscular activity [55,56]. Their concentrations might increase due to leakage into the blood from damage to liver cells, the myocardium, and skeletal muscles [57,58]. Because of these characteristics, they serve as safety indicators in both terrestrial and aquatic animals exposed to drugs and harmful substances [59,60]. After orally administering salinomycin to Korean rockfish at a water temperature of 20 °C, we observed no significant differences in ALT and AST levels post a single dose. The lack of significant changes in ALT and AST levels post salinomycin administration might suggest that this compound does not extensively damage liver cells, possibly due to the rapid metabolism of salinomycin in the liver [61]. Similar to studies in which the oral administration of albendazole in conjunction with a high-fat diet in rats limited severe elevations in liver enzymes, the co-administration of omega-3 with salinomycin might have mitigated the elevation of ALT and AST levels [62].

BUN, or blood urea nitrogen, provides an indirect evaluation of kidney function [63]. Elevated BUN levels can indicate kidney dysfunction [64]. In fish, BUN levels help detect potential impairments in the gills and kidneys, which are primary sites for ammonia excretion [65,66,67]. After orally administering salinomycin at a water temperature of 20 °C, we noticed significant differences in BUN levels on the 14th day. Although BUN alterations can be influenced by increased protein metabolic enzymes from liver activation when concurrent increases in AST and ALT are observed, an alternate perspective to consider is metabolic clearance [68]. When a substance is introduced to the body, initial metabolic responses could lead to temporary changes in certain serological markers. As the substance undergoes metabolic processing and is cleared from the system, these markers can return to baseline levels [64]. Such an effect aligns with the tendencies observed in calves administered salinomycin, where BUN demonstrated a delayed metabolic response compared to other biochemical indicators [69]. In our study, the transient elevation in BUN levels might hint at such a metabolic response, and its recovery by day 28 indicates that effective clearance mechanisms are at play. Thus, the changes might stem from gill and kidney function rather than solely from protein metabolism [65]. The transient nature of the BUN alteration suggests that the potential damage, if any, is likely temporary.

CK-MB, an isoform of creatine kinase, is a biomarker for diagnosing myocardial infarction (MI) [70]. Elevated CK-MB serum concentrations indicate cardiac tissue injury [71]. Consequently, elevated serum concentrations of CK-MB serve as a reliable indicator of cardiac tissue injury [72]. Prior research on terrestrial animals suggests that toxic doses of salinomycin impact not only the liver, but also skeletal and cardiac muscles [60,73,74,75]. Unlike these studies, which observed persistent enzyme elevations at toxic doses in mammals, our findings in Korean rockfish did not demonstrate such pronounced changes [76]. Specifically, CK-MB enzyme levels increased significantly when the fish were administered salinomycin at *M. sebastis*’s MEC concentration and its double. However, by the 14th day, these levels subsequently converged with the CK-MB levels of the control group, showing no statistically significant difference. This contrast hints that the MEC and its double dosage might not represent toxic doses for the Korean rockfish. Furthermore, considering the distinctions between the creatine kinase systems in fish and mammals, the observed elevation in CK-MB levels between days 3 and 7 post-administration in our study could suggest a different metabolic response from the immediate reactions observed in mammals [77]. To further elucidate these effects, a comprehensive evaluation emphasizing histological tissue stability is essential.

Contrary to previous studies on terrestrial animals like turkeys and camels, in which a toxic dose of salinomycin resulted in sustained increases in biochemical markers such as AST, ALT, BUN, and CK-MB, and higher mortality rates, our Korean rockfish study showed no mortality at 5 mg/kg and 10 mg/kg concentrations [78,79]. These findings are consistent with earlier fish studies, like in *Sparus aurata*, where a 6 mg/kg dose over 42 days neither caused mortality nor histological toxicity [80]. According to our results, the salinomycin dosages used are not toxic to Korean rockfish. Despite no observed mortality at these concentrations, more in-depth research is essential to understand transient biochemical changes. Future research should delve deeper into the specific mechanisms of how salinomycin impacts biochemical markers like BUN and CK-MB. It should also examine salinomycin’s long-term effects, including potential growth inhibition, and explore its pharmacokinetics, clearance, and other related physiological processes.

Water temperature influences the metabolism and behavior of aquatic organisms and plays a crucial role in fish [81]. *M. sebastis* is most actively infected within the Korean rockfish’s typical habitat temperature range, between 15 °C and 20 °C [4,5]. Prior research suggests that salinomycin, used to prevent coccidiosis in poultry, is most effective when administered before infection [23]. To determine salinomycin’s potential applicability for Korean rockfish, it is essential to confirm its preventive administration’s stability at both the optimal infection temperature of 20 °C and the pre-infection temperature of 13 °C. In our study, we observed enzyme level patterns at the suboptimal water temperature of 13 °C, confirming no significant changes in AST, ALT, and BUN compared to the 13 °C control group, with only a temporary change in CK-MB. The differences between 20 °C and 13 °C suggest metabolic changes based on water temperature, emphasizing the need for dosage adjustments. This opens the possibility for the prophylactic application of the treatment prior to the onset of *M. sebastis* infection during the initial stages of Korean rockfish aquaculture. However, more research is needed to validate its efficacy for preventative use.

Our study mainly highlights immediate effects, but a comprehensive understanding of its effects requires evaluating salinomycin’s long-term impacts, especially as a feed additive. This would address several environmental and health concerns raised by recent studies [52,82,83]. Although our assessment relies heavily on enzymatic activity measurements, a more accurate safety profile necessitates in-depth histological analyses and residue characterization. Furthermore, the effect of salinomycin on non-target species, broader ecosystems, and water quality remains unaddressed. Having established its effects on *M. sebastis* infection, upcoming research should ensure that control strategies consider both Korean rockfish’s aquacultural environment and practical industry applications.

## 5. Conclusions

In conclusion, this study explored *M. sebastis*’s susceptibility to salinomycin and assessed the efficacy and safety of its oral administration for treating *M. sebastis* infections in Korean rockfish. Our findings confirm that salinomycin exhibits dose-dependent efficacy against *M. sebastis* and that the evaluated oral administration approach is potentially effective and safe. We also found that water temperature plays a crucial role in treatment safety, with more stable enzyme patterns at 13 °C than at 20 °C. This research provides valuable insights for developing parasite control strategies using salinomycin in Korean rockfish, aiming to reduce adverse impacts on fish health and the environment. However, in-depth research is crucial for understanding salinomycin’s long-term impacts and potential side effects. Future studies should address this study’s limitations, such as the potential effects on non-target species and water quality, and the need for comprehensive histopathological evaluations.

## Data Availability

The data presented in this study are available upon request from the corresponding author.
